# High-Pressure Crystallization of iPP Nucleated with 1,3:2,4-bis(3,4-dimethylbenzylidene)sorbitol

**DOI:** 10.3390/polym13010145

**Published:** 2021-01-01

**Authors:** Przemyslaw Sowinski, Ewa Piorkowska, Severine A. E. Boyer, Jean-Marc Haudin

**Affiliations:** 1 Centre of Molecular and Macromolecular Studies, Polish Academy of Sciences, Sienkiewicza 112, 90 363 Lodz, Poland; epiorkow@cbmm.lodz.pl; 2 Centre for Material Forming, MINES ParisTech, PSL-Research University, UMR CNRS 7635, 1 Rue Claude Daunesse, 06904 Sophia Antipolis, France; severine.boyer@mines-paristech.fr (S.A.E.B.); Jean-Marc.Haudin@mines-paristech.fr (J.-M.H.)

**Keywords:** polypropylene, 1,3:2,4-bis(3,4-dimethylbenzylidene)sorbitol, nucleation, crystallization, high pressure, γ-form

## Abstract

1,3:2,4-bis(3,4-dimethylbenzylidene)sorbitol (DMDBS) is highly effective in nucleation of the α- form of isotactic polypropylene (iPP). However, its role in high-pressure crystallization of iPP, facilitating the formation of the γ- polymorph, has not been explored. The present paper focuses on the influence of DMDBS on nucleation of high-pressure crystallization of iPP. iPP with 0.2–1.0 wt.% of the DMDBS was crystallized under elevated pressure, up to 300 MPa, in various thermal conditions, and then analyzed by PLM, WAXD, SEM, and DSC. During cooling, crystallization temperatures (*T*_c_) were determined. It was found that under high-pressure DMDBS nucleated crystallization of iPP in the orthorhombic γ- form. As a consequence, *T*_c_ and the γ- form content increased for the nucleated iPP, while the size of polycrystalline aggregates decreased, although the effects depended on DMDBS content. The significant increase of *T*_c_ and the decrease of grain size under high pressure of 200–300 MPa required higher content of DMDBS than the nucleation of the α-form under lower pressure, possibly due to the effect of pressure on crystallization of DMDBS itself, which is a prerequisite for its nucleating activity.

## 1. Introduction

Isotactic polypropylene (iPP) crystallizes in different crystallographic forms. Under atmospheric pressure (*P*_atm_), iPP crystallizes usually in the monoclinic α-form. Crystallization of iPP in the trigonal β-form requires special nucleating agents [1] or special crystallization conditions, for instance zone solidification [2]. Copolymers of propylene with more than 10% of hexene or pentene comonomers, owing to the presence of their longer side chains, crystallize in the trigonal form termed as delta (δ) [3]. Moreover, in stereodefective iPP, the orthorhombic ε form was discovered, nucleated on the α-form lamellae [4]. The mesomorphic form occurs at very large undercooling.

This study focuses on the iPP crystallization in the orthorhombic γ-form. The γ-form lamellae are built of bilayers formed by parallel helices [5,6], whose axes in adjacent bilayers are inclined at approximately 80° to each other [5,6,7] and tilted at approximately 40° to the lamellar normal. The γ-phase was observed in iPP of very low molar mass [8,9,10], and also in propylene copolymers with a small content of 1-olefine co-units [11,12,13,14,15,16,17], as well as in metallocene iPP of high molar mass with stereo- or regio- chain irregularities [18,19,20,21,22]. That is explained as an enforcement of crystallization of relatively short isotactic sequences in the extended form by chain defects [20]. The requirement for chain-folding is reduced by the tilt of helix axes with respect to the lamellae normal, thus the factors impeding the chain folding facilitate crystallization of iPP in the γ-modification [21]. The effect of stereo- and regio- defects is similar [23] because the effective average length of isotactic sequences is crucial for the formation of the γ-polymorph [24,25]. Thus, the content of the γ-phase increases with increasing content of co-units and chain irregularities [12,17,20] although the distribution of defects along the chain is a very significant factor too.

The γ-phase crystallization is favored under elevated pressure (*P*) [26,27,28,29], even in highly isotactic iPP, regardless of its molar mass. Polymers, including iPP, are subjected to elevated *P* during widely used injection molding. It is worth mentioning that iPP crystallized under high *P* in the γ-form can exhibit higher modulus of elasticity and yield strength than the same polymer crystallized in the α-form [30,31]. Mezghani and Phillips [29] proposed the phase diagram for the formation of the α- and γ-form based on their extensive experimental work and comparison of the Gibbs free energy of both polymorphs. The crystallization in the γ-phase requires not only high *P,* but also high crystallization temperature, that is a small undercooling. According to [29] the equilibrium melting temperature (*T*_m_^0^) of the γ-form increases with increasing *P*, from 186.5 °C under *P*_atm_ to 241.1 °C under 200 MPa, whereas the temperature above which exclusively γ-phase forms is between 170 and 180 °C in this *P* range. Thus, the undercooling at which γ-phase forms increases with increasing *P* and the γ-domain broadens. Moreover, it was found that α-nucleating agents could increase γ-phase content in the copolymers of propylene with ethylene and 1-hexene cooled under *P*_atm_ [32,33,34] and also in iPP homopolymer [32], although the effect depended on the cooling rate.

The search for nucleating agents of iPP is an old story. Since the early works of Beck [35] and Binsbergen [36], a huge number of systems have been tested. In a recent article, Gahleitner et al. [37] reviewed the known systems for iPP nucleation. Two physically distinct groups of nucleating agents for iPP exist: particulate systems and soluble systems. 1,3:2,4-bis(3,4-dimethylbenzylidene)sorbitol (DMDBS), an efficient nucleating agent for iPP, belongs to the second group. A maximum increase in the crystallization temperature of DMDBS nucleated iPP was observed in compositions containing between 0.2 and 1.0 wt.% of the nucleant [38]. In that DMDBS concentration range, the molten iPP and the additive formed homogeneous liquid. Upon cooling, DMDBS crystallized prior to iPP without a preceding separation into two liquids and provided active nucleation sites for iPP crystals. On the contrary, in the very low concentration range, iPP crystallized prior to the additive, and the latter did not affect substantially the crystallization of the polymer.

Yang et al. [39] demonstrated that under high *P,* DMDBS enhanced the crystallization of γ- form in the impact-resistant polypropylene copolymer of a complex structure, consisting of iPP homopolymer, an amorphous ethylene–propylene random copolymer, semicrystalline ethylene–propylene copolymers with different sequence lengths, and a few of polyethylene homo-polymer. The authors concluded that under relatively low *P,* the crystalline structure was determined mainly by DMDBS, resulting in the formation of large amounts of α-phase whereas under higher *P*, the effect of *P* dominated, leading to the formation of large amounts of γ-phase.

Our previous works revealed that high pressure γ-form of iPP was nucleated on heterogeneities, which under *P*_atm_ nucleated the usual α-form [40]. Moreover, it was found that the nucleating agents, which nucleated the common α-form under *P*_atm_, efficiently nucleated the crystallization in the γ-form under high *P* [40,41,42,43]. Three nucleants were tested: particles of poly(tetrafluoroethylene), which is known to nucleate the α-form [44,45], and two commercial α-form nucleants: Hyperform HPN-20E (Milliken Chemical), containing calcium salt of cis-1,2-cyclohexanedicarboxylic acid, and ADK Stab NA-11UH (Adeka Palmarole) containing sodium 2,2′-methylene-bis-(4,6-di-t-butylphenyl) phosphate. Under high *P* these nucleants efficiently nucleated crystallization of iPP in the γ-from, which was reflected in a strong decrease of sizes of polycrystalline aggregates and in an increase of crystallization temperature during cooling. When the crystallization of iPP was not completed in the γ-domain, the process continued in the α-form during subsequent cooling. Under such conditions, the tested nucleants increased the γ-content because of their ability to enhance the crystallization in the γ-domain. The detailed studies of lamellar structure of nucleated iPP crystallized under high *P* of 200 and 300 MPa in the γ-form revealed that predominantly the γ-lamellae were nucleated through epitaxy on α-lamellae, which formed first on nucleant grains [43].

Our study focuses on high-pressure crystallization of iPP homopolymer nucleated with DMDBS. Samples of iPP nucleated with 0.2–1.0 wt.% of DMDBS were crystallized under various conditions. The effect of the nucleant on nonisothermal crystallization temperature was determined and the structure of crystallized iPP was analyzed by wide angle X-ray diffraction (WAXD), polarized light microscopy (PLM), scanning electron microscopy (SEM), and differential scanning calorimetry (DSC). The results evidenced the efficient nucleation of crystallization of iPP in the γ-form by DMDBS under high *P*. The ability of DMDBS to nucleate high-pressure crystallization of iPP resulted in an increase of crystallization temperature and a dramatic decrease of sizes of γ-polycrystalline aggregates. However, to increase crystallization temperature and decrease grain size under 200–300 MPa, larger concentration of the nucleant was necessary than that to obtain such effects through the nucleation of the α-phase under *P*_atm_.

## 2. Materials and Methods

### 2.1. Materials

iPP 3250 MR1 (PP) provided by Atofina (now Arkema, Colombes, France) with *M*_n_ of 42 kg/mol, *M*_w_ of 213 kg/mol, and isotacticity index of 0.97, was used in the study. Melt flow index, determined according to ISO 1133 under 230 °C/2.16 kg, was equal to 25 g/10 min. The same polymer was used in our previous studies [41,42,43]. DMDBS Millad 3988i from Milliken Chemical (Spartanburg, SC, USA) was used to nucleate PP. The chemical structure of DMDBS is shown in Figure S1 in SM (Supplementary Material). PP was mixed with Millad 3988i in a Brabender batch mixer at 195 °C, at 60 rpm for 10 min. Neat PP, without the nucleant, was processed in the same way. The nucleated PP samples with 0.2, 0.4, 0.6 and 1.0 wt.% of Millad 3988i are referred to through this paper as PP/M02, PP/M04, PP/M06, and PP/M1, respectively.

### 2.2. High Pressure Crystallization

High-pressure crystallization was carried out in a custom-built cell, which was described previously [30,40,46]. Specimens in the cell were compressed by an Instron tensile testing machine (Instron Corp., High Wycombe, UK) along the cell axis, at a cross-head speed of 2 mm/min. The hydrostatic *P* and temperature inside the cell were controlled with an accuracy of ±0.5 MPa and 1 °C, respectively.

To ensure good thermal contacts inside the cell, low *P* of 1.3 MPa was applied at first. Next, the specimens were heated to 250 °C and held at this temperature for 5 min, then *P* was increased to 50–300 MPa, and the specimens were cooled under this *P* to 50 °C (protocol I). Experiments under low *P* of 1.3 MPa were also carried out for comparison. In addition, selected specimens were cooled from 250 to 200 °C, *P* was increased to 200 or 300 MPa and the specimens were kept under these conditions for 1 and 4 h, or 15 min, respectively, and next cooled to 50 °C (protocol II). When the temperature of 50 °C was reached, *P* was released. The crystallized samples were analyzed ex-situ by WAXD, PLM, SEM and DSC.

To monitor the nonisothermal crystallization under high *P*, the cross-head displacement was recorded during cooling and differentiated with respect to time. In each case, the time dependence of the displacement rate exhibited a peak due to specimen volume change during crystallization. Simultaneous temperature measurement during cooling allowed to determine the temperature, at which the maximum of volume change rate occurred, which was taken as the crystallization temperature (*T*_c_), as described previously [42]. Moreover, temperature measurements during cooling showed that the cooling from 200 °C to 50 °C, where crystallization could be expected, was nearly linear with an average rate of 8 °C/min.

### 2.3. Characterization

To characterize their melting behavior, specimens were heated at 10 and 30 °C/min from room temperature to 250 °C. To confirm the nucleating efficiency of DMBDS, the specimens were nonisothermally crystallized. After 5 min annealing at 250 °C, they were cooled to room temperature at 10 °C/min. All experiments were conducted in DSC 2920 TA Instruments (New Castle, DE, USA).

To determine content of the crystallographic forms and crystallinity degree (*X*_c_), WAXD experiments were conducted using Panalytical Xpert’ PRO (Malvern Panalytical Ltd., Malvern, UK) diffraction system operating at 40 kV and 30 mA in the reflection mode, with CuKα radiation. The diffractograms were collected in the 2Θ range of 7–70° and then deconvoluted using WAXSFIT program [47]; exemplary deconvolution is shown in Figure S2 in SM. The contents of the γ-and α-phases in the crystalline phase, *K*_γ_ and *K*_α_, were calculated according to the equation proposed by Turner-Jones et al. [48]:

(1) $$K_{\gamma }=I{\left(117\right)}_{\gamma }{\left[I{\left(117\right)}_{\gamma }+I{\left(130\right)}_{\alpha }\right]}^{-1}$$

(2) $$K_{\alpha }=1-K_{\gamma }$$

where *I* denotes the integral intensity of the respective peak.

The crystallinity degree, X_c_, was also deduced from WAXD, with the amorphous halos also being determined by the deconvolution.

To have an insight into the structure of crystallized materials, 10 µm thick sections were microtomed and examined by PLM using a microscope (PZO, Warsaw, Poland) with a video camera. The exposed surfaces of selected samples were etched according to the procedure developed by Olley et al. [49] and used also by others [30,43]. Etched, washed, and dried specimens were sputtered with gold and studied with SEM JEOL JSM-5500LV (Tokyo, Japan) operating in high vacuum mode at an accelerating voltage of 10 kV.

## 3. Results and Discussion

### 3.1. Crystallization and Structure

**Figure 1:**
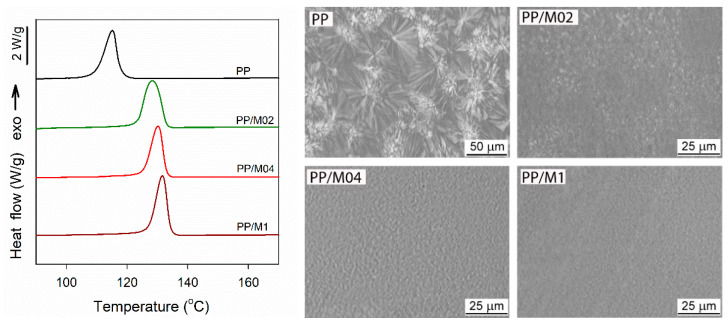
DSC cooling thermograms (**left**), and PLM micrographs of thin sections of neat and nucleated PP crystallized during cooling (**right**).

Figure 1 shows DSC cooling thermograms and PLM micrographs of thin sections of neat and nucleated PP crystallized in DSC. Crystallization exotherms of neat and nucleated PP exhibited maxima, at *T*_c_ of 116 °C and between 128–132 °C, respectively. PLM micrographs of neat PP show well-developed spherulites whereas in those of nucleated PP polycrystalline aggregates are poorly discernible because of their small size. The increase of *T*_c_ and strong decrease of grain size confirmed nucleating activity of DMBDS. It is worth noting that *T*_c_ of PP/M02, at 128 °C, was lower and grain size was larger than those of PP with higher DMDBS content.

**Figure 2:**
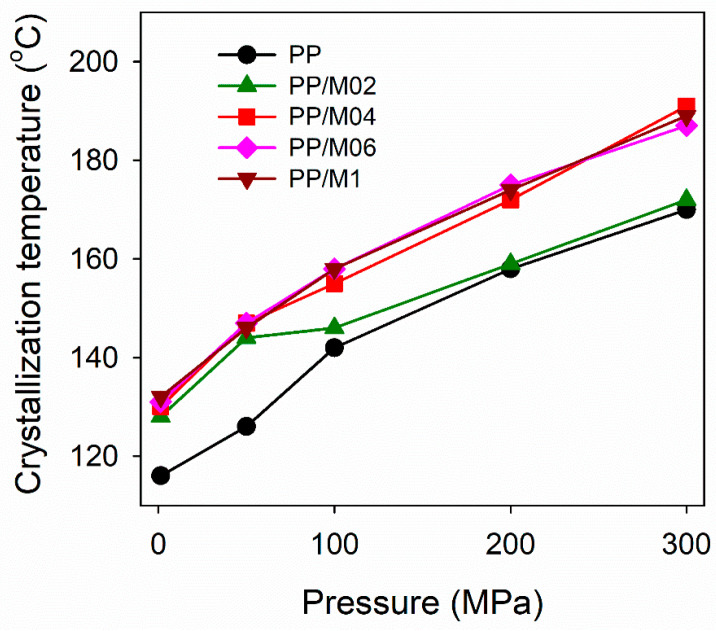
Crystallization temperature of neat and nucleated PP during cooling, according to protocol I, vs. pressure.

Figure 2 shows *T*_c_ measured for neat and nucleated PP during nonisothermal crystallization (protocol I) under various *P*. It was not possible to determine precisely cross-head displacement under 1.3 MPa, hence *T*_c_ values determined from DSC cooling thermograms are shown instead. It appears that *T*_c_ of all materials increased with increasing *P*, as can be expected, and as was observed by us previously [42], due to the increase of *T*_m_^0^. For example, according to Mezghani and Phillips [29], and Angelloz et al. [50], under 200 MPa *T*_m_^0^ of the γ-form is at 241 °C and at 232 °C, respectively, whereas linear extrapolation of their results allows to predict the rise of *T*_m_^0^ by 27 and 19 °C, respectively, with *P* increasing to 300 MPa. Regardless of *P*, *T*_c_ values of PP nucleated with 0.4–1.0 wt.% of DMDBS were similar and significantly higher than those of neat PP, by 17–21 °C under 300 MPa. *T*_c_ values of PP/M02 exceeded significantly, by up to 18 °C, those of neat PP only under relatively low *P* up to 50 MPa, and less, by only 4 °C, under 100 MPa. Under 200 and 300 MPa *T*_c_ values of PP/M02 were higher than those of PP only by 1–2 °C. Obviously, under high *P,* DMDBS nucleating activity at its 0.2 wt.% concentration was insufficient to significantly increase *T*_c_ of PP.

**Figure 3:**
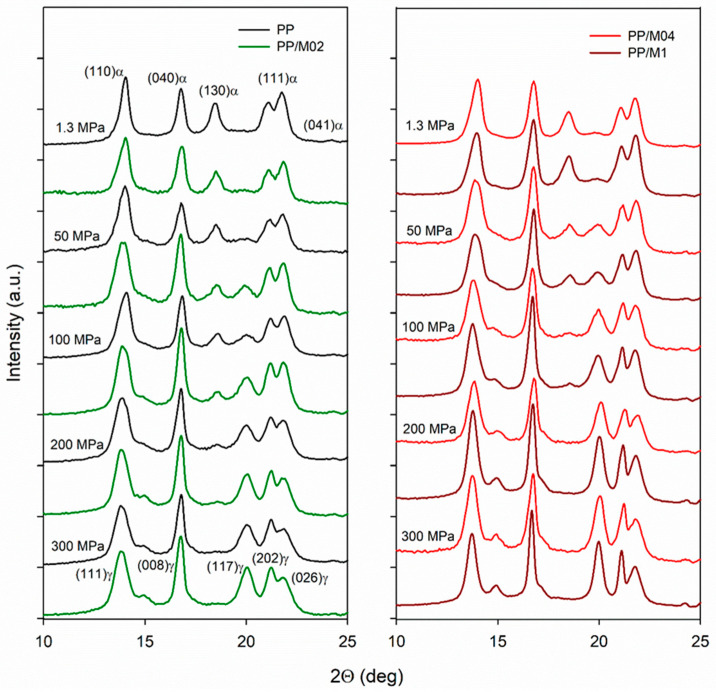
X-ray diffractograms, in 2Θ range from 10 to 25°, of neat and nucleated PP crystallized during cooling under various pressures according to protocol I.

**Figure 4:**
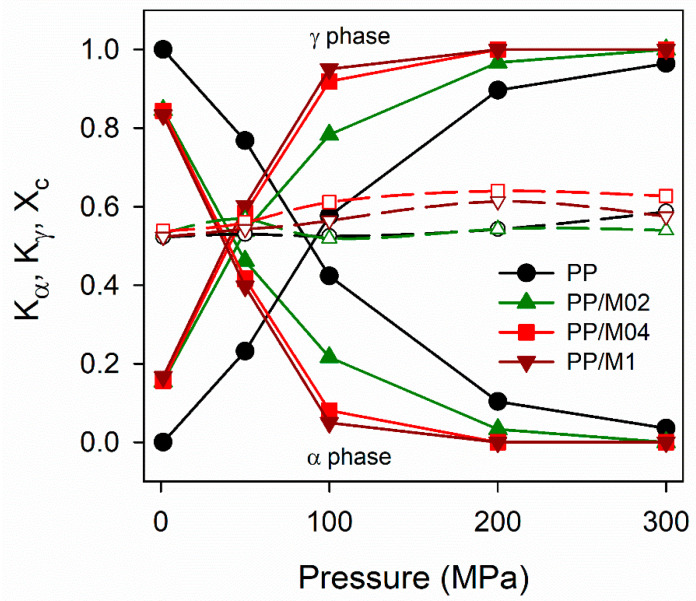
Phase contents in crystalline phase, *K*_α_ and *K*_γ_ (solid lines, filled symbols), and crystallinity degree, *X*_c_ (dashed lines, empty symbols), of neat and nucleated PP crystallized during cooling, according to protocol I, vs. pressure.

X-ray diffractograms of neat and nucleated PP crystallized under various *P* are collected in Figure 3. The diffractograms of PP/M06 are not shown because they were very similar to those of PP/M04 and PP/M1. It is seen that under 1.3 MPa, only the α-phase crystallized in neat PP, whereas in nucleated PP the predominant α-phase was accompanied by small fractions of the γ-phase, as reflected in weak (117)_γ_ peak. Under higher *P* either α- and γ-phases or pure γ-phase crystallized. The increase of *P* resulted in an increased γ-content in all materials, evidenced by the buildup of (117)_γ_ peak, accompanied by the decrease of (130)_α_ peak typical of the α-form. (130)_α_ peak was practically absent in the diffractograms of PP/M04 and PP/M1 crystallized under 200 and 300 MPa, and only a trace of it remained in the diffractograms of PP and PP/M02 crystallized under 300 MPa. Pressure dependencies of *K*_α_, *K*_γ_ and *X*_c_ are plotted in Figure 4.

In all the materials, the γ-content enlarged with increasing *P* because of broadening of the temperature range of the γ-phase formation. PP nucleated with 0.4–1.0 wt.% of DMDBS exhibited the highest γ-contents. Under 200 and 300 MPa these materials crystallized in the pure γ-form. The same applies for PP/M02 crystallized under 300 MPa, whereas under 200 MPa a trace of the α-phase also formed in this material. In neat PP, the γ-content was smaller, at 200 MPa *K*_γ_ was approximately 0.9, and even at 300 MPa it remained below 1.0. Obviously, DMDBS enhanced crystallization of PP in the γ-form, although stronger at 0.4–1.0 wt.% content. It is worth noting that there were no large differences in *X*_c_ values, although crystallinity of nucleated PP increased slightly with increasing *P*.

**Figure 5:**
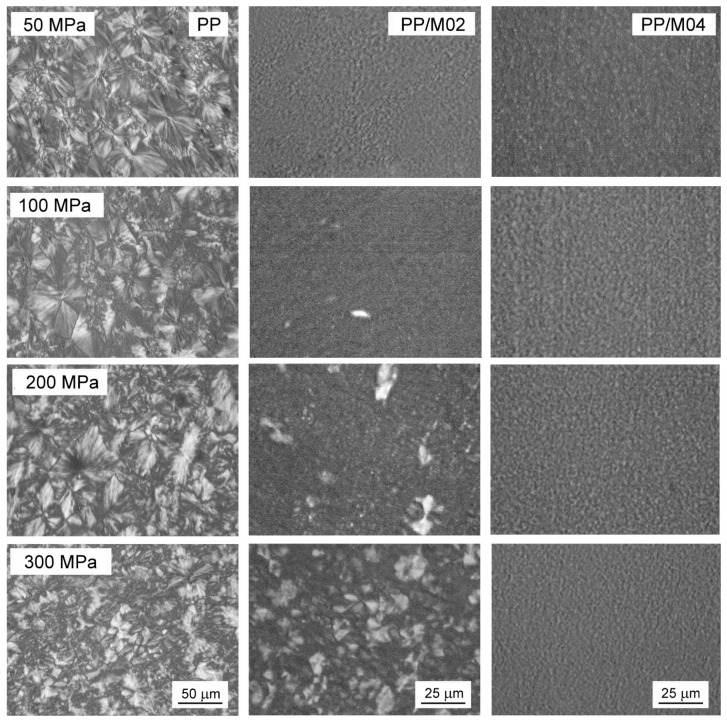
PLM micrographs of thin sections of neat and nucleated PP crystallized during cooling under various pressures according to protocol I.

**Figure 6:**
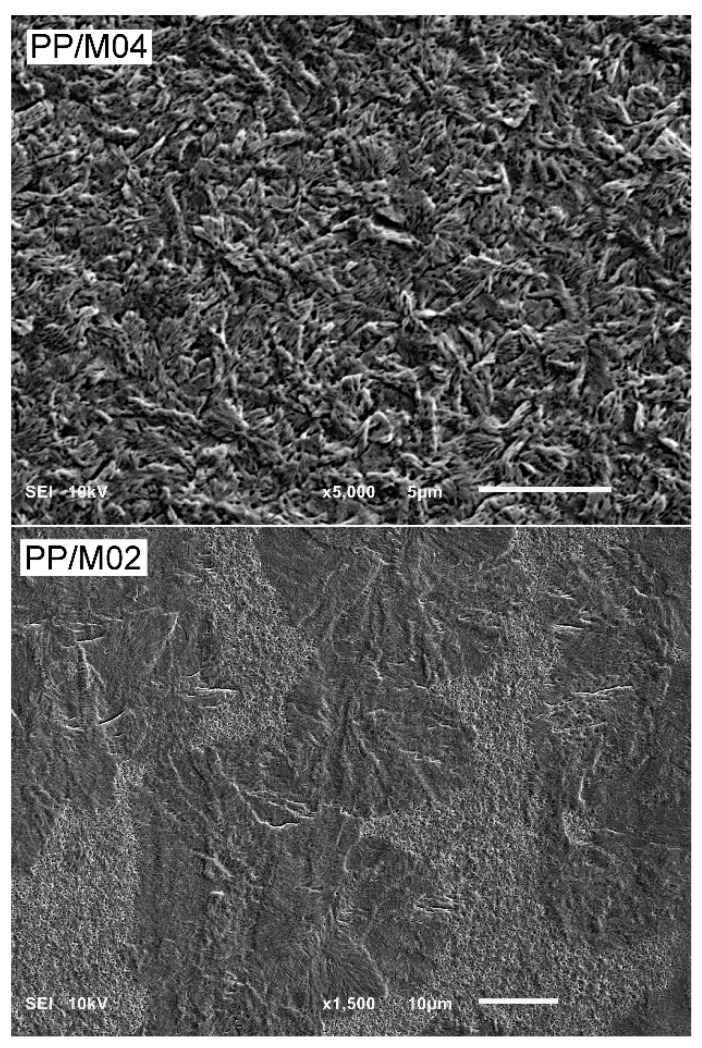
SEM micrographs of PP/M04 and PP/M02 after nonisothermal crystallization (protocol I) under 300 MPa, and etching.

PLM micrographs of thin sections of neat and nucleated PP crystallized during cooling under elevated *P* are shown in Figure 5. Polycrystalline aggregates are well visible in micrographs of neat PP crystallized in the entire *P* range, being slightly smaller at 300 MPa. According to the WAXD results the pure α-, α- and γ-, or nearly pure γ- formed in neat PP, depending on crystallization *P*. In PP/M04, the aggregates are not discernible because of their small sizes, regardless of the crystallization *P* and crystallographic form, which changed from predominant α-form under *P*_atm_ to predominant or pure γ-form under 100–300 MPa. The details of the morphology are better seen in SEM micrograph in Figure 6 showing PP/M04 permanganate etched after crystallization under 300 MPa. In the micrograph, small γ-polycrystalline aggregates are discernible, with fans composed of lamellae protruding from nucleation sites. PLM micrographs of PP nucleated with 0.6 and 1.0 wt.% of DMDBS (not shown) were similar to those of PP/M04. The PLM and SEM micrographs evidence that 0.4-1.0 wt.% of DMDBS efficiently nucleated crystallization in PP in the entire *P* range studied, either in the α- or the γ-form, depending on crystallization *P*.

In PP/M02 crystallized under 50 MPa polycrystalline aggregates are not discernible on PLM micrograph in Figure 5, due to very fine-grain structure. In this sample, the crystalline phase was composed of nearly equal parts of the α- and γ-phases, similarly to PP with higher concentrations of DMDBS and crystallized under the same *P*. Moreover, *T*_c_ of PP/M02 under 50 MPa was also similar to those of the other nucleated PP samples. Obviously, the nucleant was efficient under 50 MPa at 0.2 wt.% concentration. PLM micrograph of PP/M02 crystallized under 100 MPa shows few larger spherulites embedded in the fine-grain structure, and at 200 MPa the larger aggregates are even more numerous. At 300 MPa the structure is composed of well seen spherulites, with fine grains between them. More details of the structure formed under 300 MPa is shown in Figure 6 in SEM micrograph of PP/M02 etched after the crystallization. Although the presence of the fine grains evidenced nucleating activity of DMDBS, the large spherulites were obviously nucleated first, at higher temperature than the fine grains. Most possibly they were nucleated on the same nucleating heterogeneities and at the same temperature as spherulites in neat PP, without the nucleating agent. Taking into consideration the structure and also *T*_c_ values of PP/M02, only slightly higher, by 2–4 °C, than those of neat PP under 100–300 MPa, one can conclude that in this *P* range DMDBS nucleated the crystallization of PP in PP/M02 at the temperature lower than the temperature, at which heterogeneous nucleation occurred in neat PP. Moreover, the delay increased with increasing *P*, as can be judged from the increasing number and size of the large spherulites seen in PP/M02 in Figure 5.

According to [38], in iPP with 0.2–1.0 wt.% of DMDBS upon cooling under *P*_atm_ the nucleant crystallizes prior to the polymer, and provides active nucleation sites for iPP crystals. This is also evidenced by the DSC and PLM results shown in Figure 1. We hypothesize that at 0.2 wt.% concentration, during cooling under *P* above 50 MP, the temperature of DMDBS crystallization decreased below that of PP. As a result the intense nucleation of PP leading to the formation of its small grains occurred when large PP spherulites, nucleated as in neat PP, were already growing. However, only at 100 MPa the γ-content was significantly smaller than in PP nucleated with larger amounts of DMDBS. Thus, although at 0.2 wt.% content the temperature of nucleating activity of DMDBS under 200–300 MPa was lower than the temperature of usual heterogeneous nucleation in PP studied, it was sufficiently high to initiate crystallization in remaining melt in the γ-domain, resulting in the pure or almost pure γ-form.

**Figure 7:**
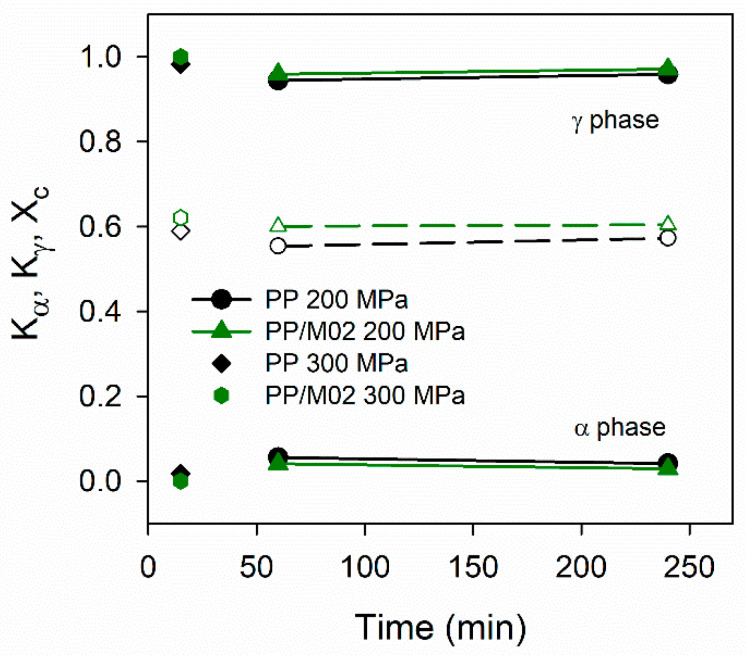
Phase contents in crystalline phase, *K*_α_ and *K*_γ_ (solid lines, filled symbols), and crystallinity degree, *X*_c_ (dashed lines, empty symbols), of neat PP and PP/M02 crystallized according to protocol II vs. dwell time at 200 °C.

The additional crystallization experiments, conducted according to protocol II, confirmed the above described observations. Figure S3 in SM compares X-ray diffractograms whereas Figure 7 shows *K*_γ_ and *K*_α_, and also *X*_c_ values of PP/M02 and neat PP held under 200 MPa at 200 °C for 1 and 4 h before subsequent cooling, and for the same materials kept at 200 °C under 300 MPa for 15 min. The diffractograms are typical of the γ-form, with pronounced (117)_γ_ peaks, whereas (130)_α_ peaks are very weak or absent. The *K*_γ_ values were high, 0.94–1.0, being slightly higher for PP/M02 than for neat PP. Under 200 MPa 1 h dwell time at 200 °C was sufficient to achieve *K*_γ_ of 0. 94–0.96. In turn, 15 min dwell time under 300 MPa resulted in *K*_γ_ of 0.98–1.0. It is worth mentioning that *X*_c_ of PP/M02 only slightly exceeded that of neat PP and weakly depended on the dwell time and *P*.

**Figure 8:**
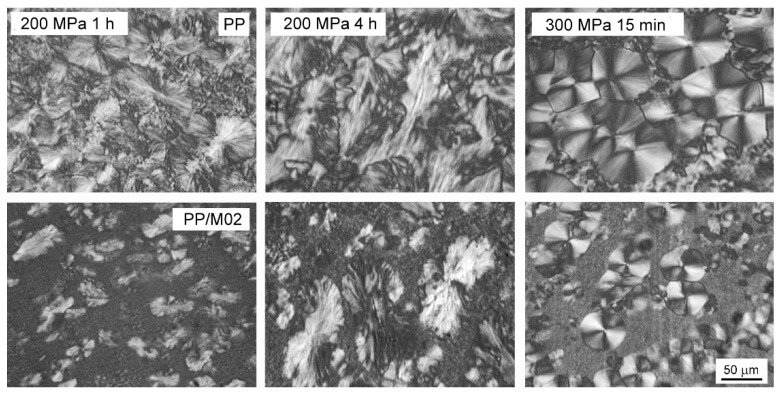
PLM micrographs of thin sections of neat PP and PP/M02 crystallized according to protocol II under 200 and 300 MPa.

PLM micrographs of thin sections of neat PP and PP/M02 crystallized according to protocol II are shown in Figure 8. In neat PP, rather large polycrystalline aggregates, which obviously started to grow at 200 °C, are accompanied by smaller ones, which grew during cooling, as described previously [41]. Obviously, the crystallization was not completed at 200 °C and continued during cooling. In turn, in PP/M02, larger polycrystalline aggregates, although smaller than in neat PP, are embedded in fine grain structure. This evidences that during cooling very intense nucleation occurred on DMDBS, and the crystallization was quickly completed by the growth of numerous and small DMDBS nucleated grains. Moreover, large spherulites differed in sizes, especially in samples kept for 4 h under 200 MPa and for 15 min under 300 MPa, which indicates that some of them could be nucleated during cooling before the occurrence of nucleation on DMDBS. These observations corroborate the conclusion on the decrease of temperature of DMDBS nucleating activity in PP/M02 under high *P*.

### 3.2. Melting

**Figure 9:**
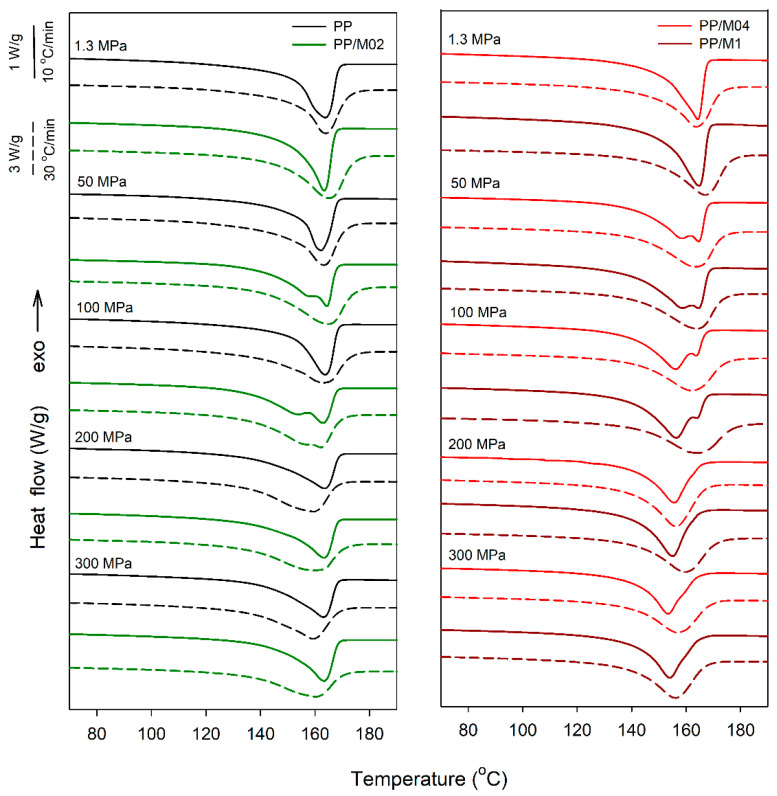
DSC heating thermograms recorded at rates of 10 °C/min (solid lines) and 30 °C/min (dashed lines) for neat and nucleated PP crystallized during cooling under elevated pressure according to protocol I.

DSC heating thermograms of nonisothermally crystallized neat and DMDBS nucleated PP are collected in Figure 9. The thermograms show the evolution from the melting peaks of the α-phase to those of the γ-phase. The melting peak temperatures (*T*_m_) of the pure or predominant α-phase formed under 1.3 MPa were located at 164–166 °C and 164–167 °C during heating at 10 and 30 °C/min, respectively.

*T*_m_ values of neat PP crystallized under elevated *P* during heating at 10 °C/min were similar, 162–164 °C, although, with increasing *P* the melting peaks broadened. During heating at 30 °C/min *T*_m_ of PP, which was crystallized under 200 and 300 MPa, decreased to 160 and 159 °C, respectively, indicating that the reorganization of the crystalline phase, predominantly in the γ-form, occurred during slower heating, as already reported by us in [42].

PP/M04 and PP/M1 crystallized under 50 MPa, during heating at 10 °C/min exhibited double-melting behavior, with *T*_m_ values near 158 and 165 °C. The double-melting behavior usually results from melting of different crystallographic forms or population of crystals differing in thickness or perfection, or from recrystallization phenomena occurring during heating. The melting temperature of the γ-phase is lower than that of the α-phase, although it can increase, for instance, due to isothermal thickening of lamellae at crystallization temperature [29]. In addition, according to the discussion of Mezghani and Phillips [29] during heating at 10 °C/min the melting of the γ-form rather occurs than its transformation to the α-form. At 100 MPa, the low-temperature melting peaks of PP/M04 and PP/M1 developed into the main peaks and their *T*_m_ values decreased to 156 °C, whereas the high-temperature peaks diminished. At 200 and 300 MPa, the high-temperature peaks vanished and only low-temperature ones remained with *T*_m_ shifted to 153–154 °C. These changes correspond to the increase of the γ-content in these materials with increasing crystallization *P*. Thus, the low- and high-temperature peaks observed at 50 and 100 MPa can be attributed to the melting of the γ- and α-form, respectively, although the reorganization in the crystalline phase cannot be entirely excluded because during faster heating only single broad melting peaks appeared, with *T*_m_ values at 162–164 °C. It is worth mentioning that at 10 °C/min only single melting peaks were observed for neat PP crystallized under 50 and 100 MPa. However, in PP the γ-content was smaller and the γ-phase crystallized at higher undercooling that in PP/M1 and PP/M04, and its reorganization during heating could affect its melting behavior.

During heating of PP/M04 and PP/M1, which were crystallized under 200 and 300 MPa, only single peaks were observed, with *T*_m_ values at 153–156 °C and 156–160 °C at 10 and 30 °C/min, respectively. Unlike in neat PP, the *T*_m_ values did not increase with decreasing heating rate, most possible due to better stability of the γ-form, which crystallized at lower undercooling.

The melting behavior of PP/M02 crystalized under 50 MPa was similar to that of PP/M04 and PP/M1, although the high-temperature peak during heating at 10 °C/min was slightly higher, possibly due to a somewhat larger α-content. However, at 100 MPa the low-temperature peak did not develop into the main melting peak, and its *T*_m_ decreased to 153 °C, most possibly because of the smaller content of the γ-phase, its lower *T*_c_, and reorganization phenomena in the crystalline phase. At 30 °C/min only broad high-temperature peaks remained, with *T*_m_ of 162 °C, however, with a small low-temperature shoulder. In turn, the thermograms of PP/M02 crystallized under 200 and 300 MPa were similar to those of neat PP, in agreement with similar *T*_c_ values of these materials during cooling.

**Figure 10:**
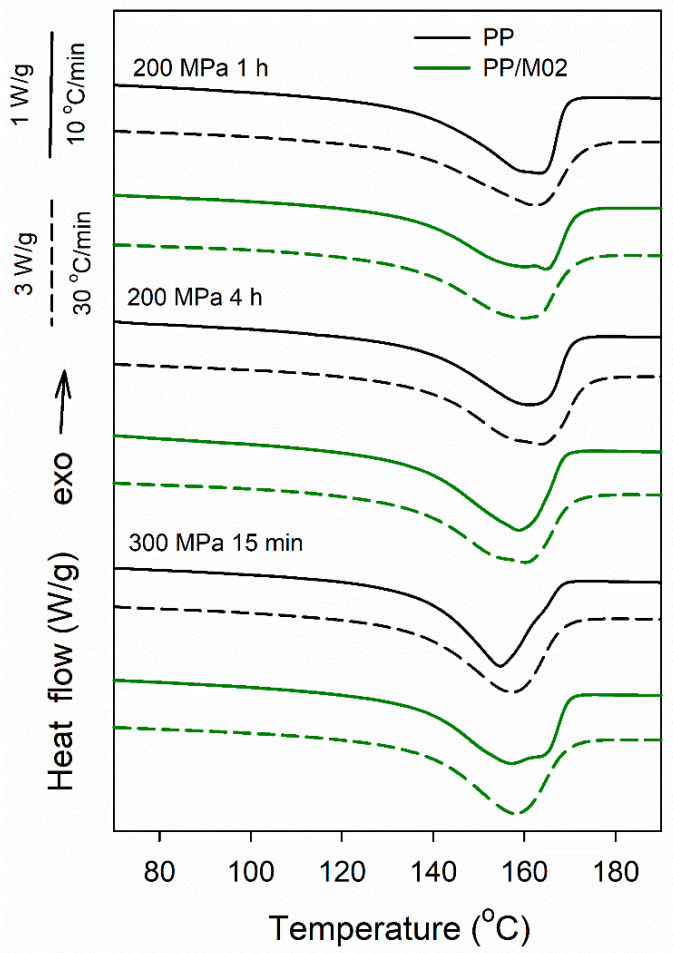
DSC heating thermograms recorded at rates of 10 °C/min (solid lines) and 30 °C/min (dashed lines) for neat PP and PP/M02 crystallized according to protocol II.

DSC heating thermograms of PP and PP/M02 crystallized under 200 and 300 MPa according to protocol II, plotted in Figure 10, show broad melting peaks of predominant or pure γ-phase, with *T*_m_ of 155–165 °C and 157–163 °C at 10 and 30 °C/min, respectively. The melting behavior reflects crystallization conditions of these materials. According to PLM results, in all these materials the crystallization started at 200 °C and was accomplished during cooling. Moreover, in PP/M02 during cooling strong nucleation on DMDBS occurred. Some thermograms recorded at 10 °C/min, shown in Figure 10, exhibited shoulders, most possibly related to the fraction of crystals formed during cooling and to reorganization phenomena in the crystalline phase, as the high-temperature parts of the peaks decreased and the shoulders disappeared during faster heating. It is also worth noting that the *T*_m_ values of PP and PP/M02 measured at 10 °C/min, were 155 and 157 °C respectively, after crystallization under 300 MPa, and higher, 159–165 °C after crystallization under 200 MPa. Most probably this is because at 200 °C the undercooling under 300 MPa is higher than under 200 MPa.

## 4. Conclusions

The influence of DMDBS on nucleation of high-pressure crystallization of iPP was studied. PP with 0.2–1.0 wt.% of the DMDBS was crystallized under elevated *P*, up to 300 MPa, in various thermal conditions. The obtained results evidence the ability of DMDBS to nucleate crystallization of PP under high *P* in the γ-form. In the entire *P* range studied, after nonisothermal crystallization (protocol I) the γ-content in the nucleated PP exceeded that in the neat PP, although it was smaller in PP/M02 than in the other nucleated materials. In the entire *P* range at 0.4–1.0 wt.% nucleant content *T*_c_ was significantly higher and a fine-grain structure formed. In PP/M02, the marked increase of *T*_c_ and the fine-grain structure was observed at 50 MPa. At 100 MPa *T*_c_ increased only slightly and a few relatively large spherulites were observed in this material embedded in the fine-grain structure. With increasing *P* the number of large spherulites increased and they became predominant at 300 MPa, although still surrounded by fine grains. Moreover, under 200 and 300 MPa *T*_c_ of PP/M02 was nearly the same as that of neat PP. Hence, it was concluded that at 0.4–1.0 wt.% concentration of DMDBS, the nucleation on it occurred at elevated temperature, whereas at 0.2 wt.% concentration DMDBS nucleated PP crystals at temperature lower than that of usual heterogeneous nucleation in this polymer. Nevertheless, the nucleation on DMDBS occurred in PP/M02 at sufficiently high temperature to promote the crystallization in the γ-domain. Hence, the γ-content increased in that *P* range, in which crystallization in the neat PP was not accomplished in γ-domain and continued in the α-form. The decrease of temperature of DMDBS activity at its 0.2 wt.% concentration under high *P* was corroborated by the results of additional studies, on crystallization of PP and PP/M02 under 200 or 300 MPa, which began in isothermal conditions at 200 °C, and was completed during cooling. In PP/M02, strong nucleation occurred on DMDBS during cooling. As a result, large spherulites nucleated at 200 °C were surrounded by fine grains nucleated on DMDBS.

The melting behavior of nonisothermally crystallized PP/M04 and PP/M1 was similar, and differed from that of PP. It reflected their high γ-content increasing with increasing crystallization *P*. Unlike for PP, no decrease of *T*_m_ with increasing heating rate was observed, evidencing better stability of the γ-phase due to its crystallization at lower undercooling. The melting of PP/M02 crystallized under 50 and 100 MPa bore some similarities to that of PP/M04 and PP/M1. In turn, the melting behavior of PP/M02 crystallized under 200 and 300 MPa resembled that of neat PP, in agreement with nearly the same *T*_c_ values of these materials. DSC heating thermograms of PP and PP/M02, which were crystallized under 200 and 300 MPa initially at 200 °C and then during cooling, reflected their complex crystallization conditions. Shoulders were observed on some thermograms recorded at 10 °C/min, which were most possibly related to the fraction of crystals formed during cooling and to reorganization phenomena in the crystalline phase, as the high-temperature parts of the peaks decreased and the shoulders disappeared during faster heating.

The obtained results show that DMDBS efficiently nucleates crystallization of PP in the γ-form under high *P*. When added in the appropriate amount, DMDBS increases *T*_c_ and the γ-content, and simultaneously reduces the grain size in the course of nonisothermal crystallization during cooling under high *P*. This indicates the possibility to use it to increase efficiently the γ-content or even to obtain the pure γ-PP during industrial processing like injection molding.

## Data Availability

The data presented in this study are available on request from the corresponding author.

## Supplementary Materials

The following are available online at https://www.mdpi.com/2073-4360/13/1/145/s1, Figure S1. Chemical structure of 1,3:2,4-bis(3,4-dimethylbenzylidene)sorbitol, Figure S2. Example of deconvolution of the diffractogram, in 2Θ range from 10 to 30°, recorded for the specimen containing both α- and γ- crystallographic forms, Figure S3. X-ray diffractograms, in 2Θ range from 10 to 25°, of neat PP and PP/M02 crystallized according to protocol II.

## References

[1] Varga J., Karger-Kocsis J. (1993). Crystallization, melting and supermolecular structure of isotactic polypropylene. Polypropylene: Structure, Blends and Composites.

[2] Lovinger A.J., Chua J.O., Gryte C.C. (1977). Studies on the α and β forms of isotactic polypropylene by crystallization in a temperature gradient. J. Polym. Sci. Polym. Phys. Ed..

[3] De Rosa C., Auriemma F., Corradini P., Tarallo O., Dello Iacono S., Ciaccia E., Resconi L. (2006). Crystal structure of the trigonal form of isotactic polypropylene as an example of density-driven polymer structure. J. Am. Chem. Soc..

[4] Lotz B. (2014). A new ε crystal modification found in stereodefective isotactic polypropylene samples. Macromolecules.

[5] Bruckner S., Meille S.V. (1989). Non-parallel chains in crystalline γ-isotactic polypropylene. Nature.

[6] Meille S.V., Bruckner S., Porzio W. (1990). γ-isotactic polypropylene—A structure with nonparallel chain axes. Macromolecules.

[7] Bruckner S., Meille S.V., Sozzani P., Torri G. (1990). Gamma-isotactic polypropylene—An MAS 13C NMR-study of a crystalline polymer with nonparallel chains. Makromol. Chem. Rapid Commun..

[8] Lotz B., Graff S., Wittmann J.C. (1986). Crystal morphology of the γ-(triclinic) phase of isotactic polypropylene and its relation to the α-phase. J. Polym. Sci. Part B Polym. Phys..

[9] Morrow D.R., Newman B.A. (1968). Crystallization of low-molecular-weight polypropylene fractions. J. Appl. Phys..

[10] Kojima M. (1967). Solution-grown lamellar crystals of thermally decomposed isotactic polypropylene. J. Polym. Sci. Part B Polym. Lett..

[11] Turner-Jones A. (1971). Development of the γ-crystal form in random copolymers of propylene and their analysis by DSC and X-ray methods. Polymer.

[12] Guidetti G.P., Busi P., Giulianelli I., Zannetti R. (1983). Structure properties relationships in some random copolymers of propylene. Eur. Polym. J..

[13] Busico V., Corradini P., De Rosa C., Di Benedetto E. (1985). Physico-chemical and structural characterization of ethylene-propene copolymers with low ethylene content from isotactic-specific Ziegler-Natta catalysts. Eur. Polym. J..

[14] Avella M., Martuscelli E., Della Volpe G., Segre A., Rossi E., Simonazzi T. (1986). Composition-properties relationships in propene-ethene random copolymers obtained with high-yield Ziegler-Natta supported catalysts. Makromol. Chem..

[15] Marigo A., Marega C., Zannetti R., Paganetto G., Canossa E., Coletta F., Gottardi F. (1989). Crystallization of the γ-form of isotactic poly(propylene). Makromol. Chem..

[16] Mezghani K., Phillips P.J. (1995). γ-Phase in propylene copolymers at atmospheric-pressure. Polymer.

[17] Hosier I.L., Alamo R.G., Lin J.S. (2004). Lamellar morphology of random metallocene propylene copolymers studied by atomic force microscopy. Polymer.

[18] Fischer D., Mulhaupt R. (1994). The influence of regio- and stereoirregularities on the crystallization behavior of isotactic poly(propylene) prepared with homogeneous group IVa metallocene/methylaluminoxane Ziegler-Natta catalysts. Macromol. Chem. Phys..

[19] Thomann R., Wang C., Kressler J., Mulhaupt R. (1996). On the γ-phase of isotactic polypropylene. Macromolecules.

[20] Alamo R.G., Kim M.H., Galante M.J., Isasi J.R., Mandelkern L. (1999). Structural and kinetic factors governing the formation of the γ polymorph of isotactic polypropylene. Macromolecules.

[21] Thomann R., Semke H., Maier R.D., Thomann Y., Scherble J., Mulhaupt R., Kressler J. (2001). Influence of stereoirregularities on the formation of the γ-phase in isotactic polypropene. Polymer.

[22] De Rosa C., Auriemma F., Circelli T., Waymouth R.M. (2002). Crystallization of the α and γ forms of isotactic polypropylene as a tool to test the degree of segregation of defects in the polymer chains. Macromolecules.

[23] De Rosa C., Auriemma F., Paolillo M., Resconi L., Camurati I. (2005). Crystallization behavior and mechanical properties of regiodefective, highly stereoregular isotactic polypropylene: Effect of regiodefects versus stereodefects and influence of the molecular mass. Macromolecules.

[24] Auriemma F., De Rosa C. (2002). Crystallization of metallocene-made isotactic polypropylene: Disordered modifications intermediate between the α and γ forms. Macromolecules.

[25] De Rosa C., Auriemma F., Spera C., Talarico G., Tarallo O. (2004). Comparison between polymorphic behaviors of Ziegler-Natta and metallocene-made isotactic polypropylene: The role of the distribution of defects in the polymer chains. Macromolecules.

[26] Mezghani K., Phillips P.J. (1997). The γ-phase of high molecular weight isotactic polypropylene. II: The morphology of the γ-form crystallized at 200 MPa. Polymer.

[27] Kardos J.L., Christiansen A.W., Baer E. (1966). Structure of pressure-crystallized polypropylene. J. Polym. Sci. Part A-2 Polym. Phys..

[28] Kalay G., Zhong Z.P., Allan P., Bevis M.J. (1996). The occurrence of the γ-phase in injection moulded polypropylene in relation to the processing conditions. Polymer.

[29] Mezghani K., Phillips P.J. (1998). The γ-phase of high molecular weight isotactic polypropylene: III. The equilibrium melting point and the phase diagram. Polymer.

[30] Lezak E., Bartczak Z., Galeski A. (2006). Plastic deformation of the γ phase in isotactic polypropylene in plane-strain compression. Macromolecules.

[31] von Baeckmann C., Wilhelm H., Spieckermann F., Strobel S., Polt G., Sowinski P., Piorkowska E., Bismarck A., Zehetbauer M. (2019). The influence of crystallization conditions on the macromolecular structure and strength of γ-polypropylene. Thermochim. Acta.

[32] Foresta T., Piccarolo S., Goldbeck-Wood G. (2001). Competition between α and γ phases in isotactic polypropylene: Effects of ethylene content and nucleating agents at different cooling rates. Polymer.

[33] Perez E., Zucchi D., Sacchi M.C., Forlini F., Bello A. (1999). Obtaining the γ phase in isotactic polypropylene: Effect of catalyst system and crystallization conditions. Polymer.

[34] Shi Q., Cai C.L., Ke Z., Yin L.G., Liu Y.L., Zhu L.C., Yin J.H. (2008). Effect of the nucleating agent 1,3: 2,4-di(3,4-dimethylbenzylidene) sorbitol on the γ phase content of propylene/ethylene copolymer. Eur. Polym. J..

[35] Beck H.N. (1967). Heterogeneous nucleating agents for polypropylene crystallization. J. Appl. Polym. Sci..

[36] Binsbergen F.L. (1970). Heterogeneous nucleation in the crystallization of polyolefins: Part 1. Chemical and physical nature of nucleating agents. Polymer.

[37] Gahleitner M., Grein C., Kheirandish S., Wolfschwenger J. (2011). Nucleation of polypropylene homo- and copolymers. Int. Polym. Proc..

[38] Kristiansen M., Werner M., Tervoort T., Smith P., Blomenhofer M., Schmidt H.W. (2003). The binary system isotactic polypropylene/bis(3,4-dimethylbenzylidene)sorbitol: Phase behavior, nucleation, and optical properties. Macromolecules.

[39] Yang G., Li X.-X., Yang J.-H., Huang T., Zhang N., Liu X.-R., Wang Y. (2013). Crystallization behavior of sorbitol derivative nucleated polypropylene block copolymer under high pressure. Colloid Polym. Sci..

[40] Zapala K., Piorkowska E., Hiltner A., Baer E. (2012). High-pressure crystallization of isotactic polypropylene droplets. Colloid Polym. Sci..

[41] Sowinski P., Piorkowska E., Boyer S.A.E., Haudin J.M., Zapala K. (2015). The role of nucleating agents in high-pressure-induced gamma crystallization in isotactic polypropylene. Colloid Polym. Sci..

[42] Sowinski P., Piorkowska E., Boyer S.A.E., Haudin J.M. (2016). Nucleation of crystallization of isotactic polypropylene in the gamma form under high pressure in nonisothermal conditions. Eur. Polym. J..

[43] Sowinski P., Piorkowska E., Boyer S.A.E., Haudin J.M. (2018). On the structure and nucleation mechanism in nucleated isotactic polypropylene crystallized under high pressure. Polymer.

[44] Masirek R., Piorkowska E. (2010). Nucleation of crystallization in isotactic polypropylene and polyoxymethylene with poly(tetrafluoroethylene) particles. Eur. Polym. J..

[45] Galeski S., Piorkowska E., Rozanski A., Regnier G., Galeski A., Jurczuk K. (2016). Crystallization kinetics of polymer fibrous nanocomposites. Eur. Polym. J..

[46] Psarski M., Piorkowska E., Galeski A. (2000). Crystallization of polyethylene from melt with lowered chain entanglements. Macromolecules.

[47] Rabiej M. (2013). Application of immune and genetic algorithms to the identification of a polymer based on its X-ray diffraction curve. J. Appl. Crystallogr..

[48] Turner Jones A., Aizlewood J.M., Beckett D.R. (1964). Crystalline forms of isotactic polypropylene. Makromol. Chem..

[49] Olley R.H., Hodge A.M., Bassett D.C. (1979). A permanganic etchant for polyolefines. J. Polym. Sci. Polym. Phys. Ed..

[50] Angelloz C., Fulchiron R., Douillard A., Chabert B., Fillit R., Vautrin A., David L. (2000). Crystallization of isotactic polypropylene under high pressure (γ phase). Macromolecules.

