# Supplementary material for: High-Pressure Crystallization of iPP Nucleated with 1,3:2,4-bis(3,4-dimethylbenzylidene)sorbitol
Source: Polymers (Basel). 2021 Jan 1;13(1):145. doi: 10.3390/polym13010145 (PMC7795768; doi:10.3390/polym13010145)
Supplement: Supplementary file 1 [file polymers-13-00145-s001.pdf]

Supplementary Material

High pressure crystallization of iPP nucleated with 1,3:2,4-bis(3,4-dimethylbenzylidene)sorbitol

Przemyslaw Sowinski[1], Ewa Piorkowska[1], Severine A. E. Boyer[2], Jean-Marc Haudin[2]

[1] Centre of Molecular and Macromolecular Studies, Polish Academy of Sciences, Sienkiewicza 112, 90 363, Lodz, Poland

[2] Centre for Material Forming, MINES ParisTech, PSL - Research University, UMR CNRS 7635, 1 Rue Claude Daunesse, 06904, Sophia Antipolis, France

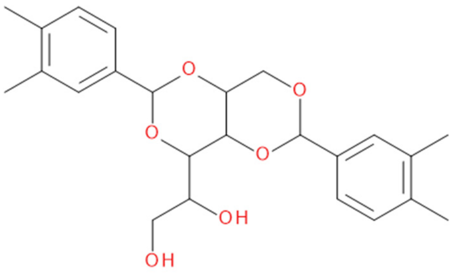

Figure S1. Chemical structure of 1,3:2,4-bis(3,4-dimethylbenzylidene)sorbitol.

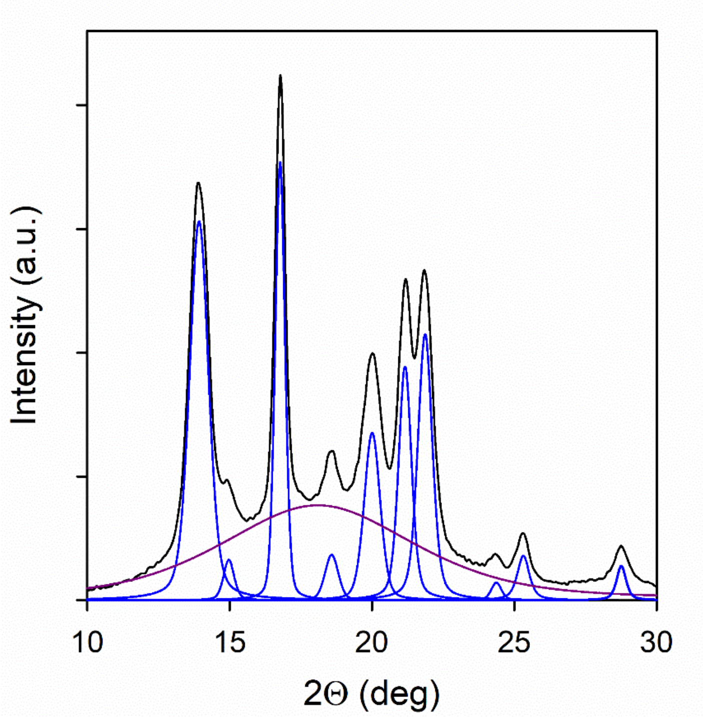

Figure S2. Example of deconvolution of the diffractogram, in 2Θ range from 10 to 30°, recorded for the specimen containing both α and γ crystallographic forms.

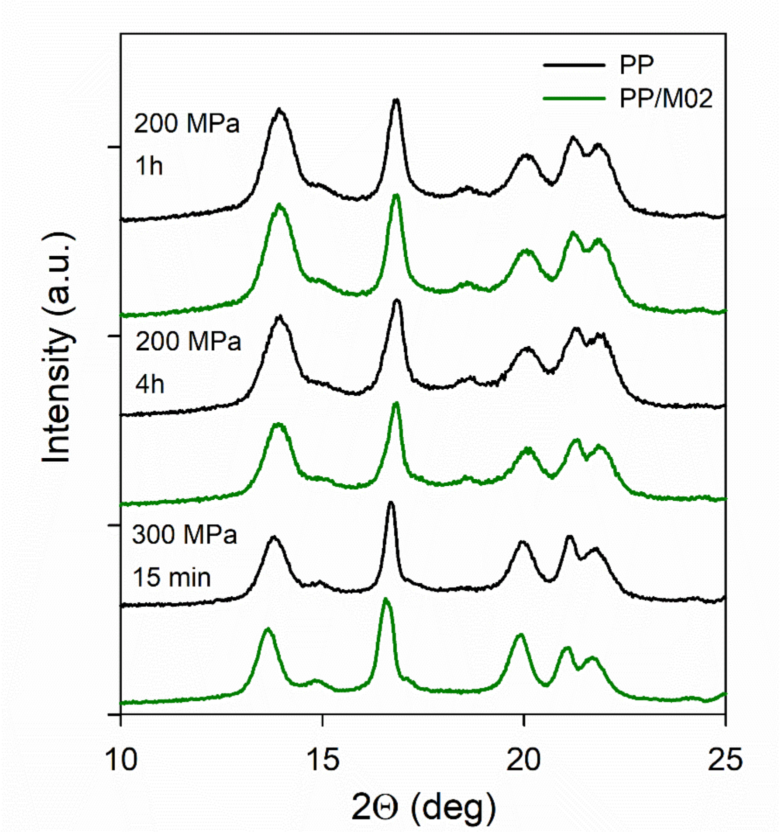

Fig. S3. X-ray diffractograms, in 2Θ range from 10 to 25°, of neat PP and PP/M02 crystallized according to protocol II.
